# Exercise therapy in primary biliary cirrhosis: the importance of moving while sitting on a surgical waiting list—a case study

**DOI:** 10.1136/flgastro-2015-100672

**Published:** 2016-04-12

**Authors:** Kate Hallsworth, Laura Jopson, David E Jones, Michael I Trenell

**Affiliations:** 1 Institute ofCellular Medicine, Newcastle University, Newcastle upon Tyne, UK; 2 Liver Unit, The Newcastle Upon Tyne Hospitals NHS Foundation Trust, Newcastle upon Tyne, UK

**Keywords:** PRIMARY BILIARY CIRRHOSIS, LIVER TRANSPLANTATION

## Abstract

**Background:**

It is being increasingly recognised that reduced cardiorespiratory fitness is associated with poorer outcomes after major surgery. Exercise limitation and reduced aerobic capacity are common in people with end-stage liver disease. There is limited evidence about the role of exercise therapy in the management of primary biliary cirrhosis (PBC) and no studies have looked at the effect of exercise in people with PBC who are awaiting liver transplantation. This case study is the first to report that personalised exercise therapy improves cardiorespiratory fitness in a patient with PBC without worsening symptoms of severe fatigue.

**Methods:**

Cardiopulmonary exercise testing was used to assess cardiorespiratory fitness in a patient with end-stage PBC prior to listing for transplantation. A personalised exercise programme was designed to improve cardiorespiratory fitness while the patient was on the transplant waiting list.

**Results:**

Anaerobic threshold, VO_2PEAK_ and maximum workload all improved with regular exercise. Fatigue levels remained unaltered.

**Conclusions:**

This patient tolerated and adhered to a personalised exercise programme for a prolonged period of time while awaiting surgery despite significant fatigue and disease burden. Liver transplantation was successfully completed and this woman remains well over 2 years post-surgery.

## Introduction

Primary biliary cirrhosis (PBC) is a chronic cholestatic liver disease with an autoimmune aetiology. A minority of patients with PBC can progress to end-stage liver disease and the need for liver transplantation. Fatigue is commonly associated with PBC and approximately 20% of patients will experience severe fatigue which can limit physical activity and often has a negative impact on quality of life. This decrease in daily physical activity results in general deconditioning and reduced cardiorespiratory fitness. Physical deconditioning can pose a problem if the patient is to be considered for liver transplantation, as cardiorespiratory fitness forms part of the assessment of anaesthetic and overall surgical risk. Patients that have better cardiorespiratory fitness prior to undergoing major hepatobiliary surgery have been shown to have improved peri-operative and post-operative outcomes, with lower complication rates.^1^ Indeed, people with low cardiorespiratory fitness are at increased risk of mortality within 100 days post liver transplantation.^2^ We trialled a period of pre-operative exercise therapy in a patient with PBC, prior to listing for liver transplantation, with the aim of improving cardiorespiratory fitness, a key biomarker of surgical morbidity and mortality. We also assessed the impact of regular exercise on patient fatigue.

## Presentation of the case

A 44-year-old woman with PBC was referred for consideration of liver transplantation due to intractable pruritus. She had been diagnosed with PBC in 2007, treated with ursodeoxycholic acid (UDCA) but suffered with severe fatigue and intractable pruritus which had been resistant to all anti-pruritic therapies including molecular adsorbent recirculating system. At the time of liver transplant assessment she was a non-responder to UDCA but bilirubin and liver synthetic function were normal, model for end-stage liver disease (MELD) was 6 and UK end-stage liver disease (UKELD) 45. The transplant assessment did not identify any contraindications to transplantation, however concern about cardiorespiratory fitness was raised as cardiopulmonary exercise test (CPET) revealed a low anaerobic threshold (AT) of 8.8 mL/kg/min, VO_2PEAK_ 13.4 mL/kg/min and maximum cycle power output of 66 watts. The multidisciplinary team decided to refer the patient to the MoveLab at Newcastle University for further exercise testing (see Hallsworth *et al*^3^ for CPET protocol) with a view to implementing a personalised home exercise programme to improve her general fitness prior to listing for transplantation.

An exercise programme was designed to focus on strengthening leg and respiratory muscles, and improving general cardiorespiratory fitness. All exercises were completed on a static bicycle in the patient's home. The exercise intensity was guided by the ‘rating of perceived exertion scale’^4^ and was based on the concept of interval training which interspersed ‘hard’ bursts of exercise with active recovery. Intervals initially lasted for 30 s with a maximum of 2 min active recovery in between. The patient tried to complete 10 hard intervals per session but this had to be adjusted according to the patient's fatigue levels on each particular day. The patient aimed to complete the programme 3–4 times/week and was contacted at regular intervals to check adherence and to modify the programme as necessary.

Physical activity levels were assessed objectively using a multisensor array (SenseWear Pro_3_, Bodymedia, Pennsylvania, USA) for 7 days prior to starting the exercise programme, and for 7 days after subsequent visits. Fatigue was assessed using the Fatigue Impact Scale.^5^ See table 1 for results.

**Table 1:** Exercise test results, fatigue and physical activity data

	CPET results			Fatigue	Physical activity data (daily means)			
	AT (mL/kg/min)	VO_2PEAK_ (mL/kg/min)	Max work rate (watts)	FIS	Steps	Energy expenditure (kcal)	Average METS	Physical activity duration (min)
April 2010*	8.8	13.4	66	–	–	–	–	–
July 2010†	9	19	86	120	5822	1722	1.3	44
September 2010	16	20	102	115	7701	1884	1.4	92
December 2010	10	21	80	–	6931	1757	1.3	48
January 2011	14	22	109	–	–	–	–	–
May 2011	10	20	105	107	5869	1727	1.3	43
July 2011	12	22	115	125	6638	1754	1.3	53

*Results from initial CPET.

†Results from second CPET prior to undertaking exercise programme.

AT, anaerobic threshold; CPET, cardiopulmonary exercise test; FIS, Fatigue Impact Scale; METS, metabolic equivalents.

## Results

AT, VO_2PEAK_ and maximum work all improved markedly from initial CPET testing (see table 1). Results fluctuated over the time course of the exercise programme depending on how the symptoms of PBC were affecting daily life and we would expect improvements to taper off as the person moves into the maintenance phase of an exercise programme. Fatigue levels did not alter. Physical activity levels remained relatively unchanged despite an increase in cardiorespiratory fitness.

Orthotopic liver transplantation was successfully carried out in August 2013 and 2 years on this woman remains well post surgery.

## Discussion

This case study demonstrates for the first time that exercise therapy can improve cardiorespiratory fitness in patients with end-stage PBC without worsening the symptoms of fatigue. This patient tolerated and adhered to a personalised exercise programme for a prolonged period of time while awaiting surgery despite significant fatigue and disease burden with physiological benefits (as demonstrated by an improvement in AT). Without ‘pre-habilitation’ to improve aerobic fitness, this woman may not have been a viable candidate for major surgery and may not have had such a positive surgical outcome. Patients should not be excluded from consideration for liver transplantation on the basis of low cardiorespiratory fitness as this can be improved with guided home exercise. Despite an increase in cardiorespiratory fitness, day-to-day physical activity levels remained unchanged and this may be due to the high levels of chronic fatigue experienced by the patient.

Future work is needed to investigate the peri-surgical and post-surgical effect of cardiorespiratory fitness levels on recovery post liver transplantation. A large-scale study is also warranted to evaluate the effectiveness and feasibility of pre-operative exercise therapy prior to liver transplantation.
